# Diet in Disease

**Published:** 1893-04-01

**Authors:** Ernest Hart

**Affiliations:** Bachelier-ès-Sciences-es-Lettres (rèstreint), formerly Student of the Faculty of Medicine of Paris, and of the London School of Medicine for Women


### DIET IN DISEASE.

By Mrs. Ernest Hart, Bachelier-ès-Sciences-es-Lettres (rèstreint), formerly Student of the Faculty of Medicine of Paris, and of the London School of Medicine for Women.

*(Continued from page 408, Vol. XIII.)*

### XVIII.—INDIGESTION.

Indigestion is the most universal of complaints. It afflicts alike the rich and the poor, those who eat too much, and those others who eat too little; the idle and the busy; the young and the old. When, however, the length and the complication of the process of digestion is considered, and when it is remembered that the slightest deflection from the normal will cause pain and discomfort, it is not surprising that indigestion is such a common complaint; still less so when it is borne in mind that in order to please the palate by agreeable flavours and sensations the average civilised man in well-to-do circumstances taxes the long-enduring powers of digestion to the very utmost. The organs concerned in the process of digestion are the mouth, the stomach, the liver, and the pancreas; disturbance or impaired action of one or any of these may be the cause of indigestion.

**Causes of Indigestion: In the Mouth.**—The food may be insufficiently masticated, owing to the teeth being decayed or deficient. If the food remains too short a time in the mouth it is not properly ground into a pulp by the grinders, and is not sufficiently mixed with the saliva. If introduced into the stomach in an unmasticated condition, the food takes a much longer time to be broken up by the action of the muscular movements of the stomach and submitted to the action of the gastric juice. The saliva being alkaline, stimulates the secretion of the acid gastric juice, and thereby exercises a considerable influence in quickening and aiding digestion; it is therefore important that the food should be well mixed with saliva before being swallowed, and that those who have a tendency to indigestion should masticate their food slowly and thoroughly.

**In the Stomach.**—The most important part of digestion takes place in the stomach. Here the food is thoroughly triturated by the movements of the muscular walls of the stomach, and mixed with the mucus and gastric juice poured out from the glands of the stomach; the albuminoids are acted upon by the pepsine, and absorbed by the veins of the stomach in the form of albuminose. The most frequent cause of stomachal indigestion is chronic gastritis. In this malady the mucous membrane of the stomach is subject to frequent attacks of sub-acute inflammation, with the result that the gastric follicles become atrophied; these cells degenerated, while the mucous glands become hypertrophied. The consequence is that the gastric juice is deficient in quantity and poor in quality, while mucus is secreted in excess. When this malady is once firmly established, it is very difficult, if not impossible, to cure The mucous membrane of the stomach may be, however, in a condition anatomically healthy; but indigestion may be caused by irregular nervous action, or by an unhealthy condition of the blood, in which case the gastric juice may be secreted in excess, giving rise to "acidity," or it may be secreted insufficiently, causing slow and difficult digestion of the albuminoids. The gastric juice may also contain either too little or too much acid. In the first case, the pepsine is slow and uncertain in action; in the second, digestion may be too rapid. These irregularities, in the secretion and condition of the gastric juice, may be due to the want of proper nervous control of the minute blood-vessels which feed the peptic glands, or to abnormal conditions of the blood, as in fevers, anæmia, diabetes, &c. Stomachal indigestion may be also caused by slow, sluggish movements of the muscular walls of the stomach, or by too rapid and energetic movements which cause the food to be ejected into the duodenum before it is thoroughly broken up and submitted to the action of the gastric juice. It will be seen from the foregoing that the causes of indigestion in the stomach are many and various, hence the extreme difficulty of treating this complaint in any well defined rule. The symptoms may be the same, but the causes are different, and must be differently treated. In one case alkalies are indicated, in another hydrochloric acid gives relief; in one food must be taken at frequent intervals, in another long periods of rest must be given; in one fluid food can alone be borne, in another the meals must be taken without drinking. The stomach has, moreover, many idiosyncrasies and antipathies which are either constitutional and permanent, or functional and temporary. Thus one person cannot digest strawberries, another cannot take onions; one person cannot eat shellfish, and mushrooms will give another an attack of indigestion. The dietetic treatment of stomachal dyspepsia can therefore be only correctly arrived at by experiment based on certain well-known principles; and, in fact, each dyspeptic must discover for himself what he can eat, how much, and how often. I am convinced that indigestion need not be such an universal complaint if people would treat their bodies in the same way as they treat their employés, and make them work hard on the lowest possible wage, with proper periods for rest. Abstemiousness and physiological rest are, in my opinion, the initial principles involved in the successful treatment of gastric dyspepsia. If the indigestion is caused by want of nervous tone, give the stomach only as much food to digest as will maintain strength; if it is caused by alcoholic excess, cut off all wines and spirits; if caused by irregularities in the production and quality of the gastric juice, rest the stomach as much as possible till nature effects her own cure. The doctrine of rest is not sufficiently considered in treating derangements of the stomach. If we have a sore and excoriated skin wound, we take care to rest the part so as to let healthy granulation go on undisturbed. The mucous membrane of the stomach may be considered in acute forms of dyspepsia to be much in the same condition, and its cure would be better accomplished by rest than by anything else. Such rest could be partially obtained by taking predigested and fluid foods; but unfortunately the practice of the dyspeptic is generally the opposite. Finding the appetite fail, and thinking it the most important of physical duties to eat, he tempts appetite and stimulates a fatigued and jaded stomach by highly spiced foods and by dainty dishes, thus often rendering chronic a condition which might have been only temporary. "Why do you come to Carlsbad?" I once asked a visitor who was a well known diner-out in London society, and who by his air of general well-being did not seem to need a "cure." "Because," was the reply, "it enables me to eat what I like for the rest of the year." This is an example of what I mean by curing dyspepsia by means of resting the stomach. The plentiful ablutions of the mucous membrane, and the rigid abstemiousness of life and diet insisted upon at Carlsbad and other spas give the stomach the chance and opportunity of curing its dyspepsia by rest.

**Duodenal Indigestion.**—After the food has been acted upon by the gastric juice in the stomach, and the albuminoids have been in a great measure rendered soluble and absorbed, the acid chyme passes into the duodenum, where it is acted upon by the bile and the pancreatic juice, both of which are alkaline. The rôle of the bile is to emulsify the fats, and of the pancreatic juice to turn the starch into glucose. The bile is secreted by the liver, and it is rightly thought that the liver plays a large part in causing indigestion. If the bile is deficient there is constipation and often distressing flatulence; if it is secreted in excess the bile salts are not reabsorbed, but circulate in the blood, producing great depression of spirits. The bile may also regurgitate into the stomach, causing vomiting of a very acrid substance. If the pancreas fails to fulfil its part, the starchy foods are not converted into glucose, the process of digestion is not completed, and undigested food passing down the alimentary canal causes irritation and diarrhœa, and the patient emaciates.

How these varying causes and conditions of indigestion can be combated by diet will be the next subject of consideration.

